# Web review: Contrast media in radiology and imaging

**Published:** 2009-11

**Authors:** IK Indrajit

**Affiliations:** Command Hospital (Air Force), Bangalore – 560 007, India

Few web-based sites related to contrast media in radiology and imaging, available at the moment, are reviewed below.

1. **Introduction to Intravenous Radiographic Contrast** is a concise summary of the subject by Romell Dhadha. Featured as a part of the Department of Radiology Short Clerkship: Lectures at University of Iowa, the material is available at http://www.radiology.uiowa.edu/Radshortclerkship/RadShortClkship/LectureNotes/Dhada.htm.
2. **A Simple Guide to the Basics of Contrast Media** is available in the form of Guide notes at http://www.e-radiography.net/contrast_media/contrast_media_introduction.htm. The material includes iodinated intravascular contrast agents, barium sulfate, computerized tomography (CT) contrast agents, biliary contrast agents and reactions, allergic emergencies.
3. From **eMedicine**, comprehensive educative material on radiographic iodinated contrast media is obtainable at http://www.emedicine.com/radio/byname/contrast-medium-reactions-recognition-and-treatment.htm.
4. The webportal **Chest Xray.com** deals concisely with radiology contrast at http://www.chestx-ray.com/Practice/Contrast.html. The following sections are covered: general, non-ionic vs ionic, risk factors, severity of reactions, renal toxicity, screening creatinine and treatment. Incidentally, the portal chest Xray.com at http://www.chestx-ray.com/ covers areas related to research, education, practice and resources.
5. **Contrast Media in Diagnostic Radiology** from the **Textbook of Radiology** at http://www.medcyclopaedia.com/library/radiology/chapter07.aspx is an up-to-date feature from Medcyclopaedia™. Medcyclopaedia™ is a “unique combination of a scientific library and a handy toolbox on the internet.” In addition, the site features the complete online edition of The Encyclopaedia of Medical Imaging, the complete online edition of A Global Textbook of Radiology, GE Healthcare's Expanded Medical Imaging Glossary, clinical cases for training purposes and a complete e-learning solution in normal imaging anatomy.
6. A **Symposium on Ultrasound Contrast for Radiological Diagnosis** titled **Bubbles in Radiology - The State of the Art** is available at http://www.sunnybrook.utoronto.ca/bubble/. The site requires a mandatory registration. Additionally, ‘A Handbook of Contrast Echocardiography’ authored by Harald Becher and Peter N Burns is available at http://www.sunnybrook.utoronto.ca/EchoHandbook/. Within this, there is an Interactive Glossary of Contrast Ultrasound at http://www.sunnybrook.utoronto.ca/EchoHandbook/bookindex.htm.
7. **Controversies and Consensus in Imaging and Interventions** is a journal for medical imaging professionals, focusing on current issues in radiology, imaging and interventional radiology/cardiology and CT radiology. This website, sponsored by an educational grant from GE Healthcare, has a section on contrast media that is available at http://www.c2i2.org/contrast_media.asp.
8. An educative supplement on **Issues in Contrast and CT Angiography with Multislice CT** is featured in Applied Radiology (Mar 2002), accessible at http://www.appliedradiology.com/backissues/issue.asp?ID=71, after a compulsory registration. The material covers important topics such as optimizing contrast use in multislice CT, fast CT in neurological imaging: contrast issues, contrast multiplies cardiac CT applications, CTA of extremities: new approaches to scanning, contrast use, new contrast administration protocols: safety considerations. A panel discussion is also available at the end.
9. Guidelines and manuals on the use of contrast media in radiology are issued periodically from reputed professional bodies. Currently available comprehensive references include
  - **American College of Radiology Contrast Manual on Contrast Media** version 6/2008, which is available at http://www.acr.org/SecondaryMainMenuCategories/quality_safety/contrast_manual.aspx.
  - **ACR Practice Guideline for the Use of Intravascular Contrast Media**, which is accessible at http://www.acr.org/s_acr/bin.asp?CID=541andDID=12241andDOC=FILE.PDF.
  - **European Society of Urogenital Radiology (ESUR) Guidelines on Contrast Media**, available from http://www.esur.org/fileadmin/Guidelines/ESUR_2007_Guideline_6_Kern_Ubersicht.pdf.
  - **Royal College of Radiology Standards for Iodinated Intravascular Contrast Agent Administration to Adult Patient**, available from http://www.rcr.ac.uk/docs/radiology/pdf/IVcontrastPrintFinal.pdf.
10. **Review Articles/Citations on Contrast media in Radiology:** There are numerous articles on contrast media in radiology available from various journals. From these, a handy ‘must read’ list of review articles on this subject, published in recent times and available online, is given below in Table 1.

**Table 1:** Review articles/citations on contrast media in radiology

Sr no.	Article/citation title	Author (s)	Year	Online detail of journal with URL
a)	Guidelines for Contrast Media from the Hiropean Society of Urogenital Radiology	Thomsen FJS	2003	http://www.ajronline.org/cgi/reprint/18l/6/1463.pdf
b)	Contrast media and the kidney: European Society of Urogenital Radiology (ESUR) Guidelines	Thomsen HS, Morcos SK	2003	http://bjr.birjournals.org/cgi/reprint/76/908/513
c)	Contrast Nephropathy: Review Reusing on Prevention	Maeder M *et al.*	2004	http://content.onlinejacc.Org/cgi/reprint/44/9/1763
d)	Contrast-induced nephropathy	Gleeson TG	2004	http://www.ajronline.Org/cgi/reprint/183/6/1673
e)	Frequently Asked Qiestions: lodinated Contrast Agents Current Practice Issues	Bettmann MA	2004	http://radiographics.rsnajnls.org/cgi/content/full/24/suppl_1/S3
f)	Acute serious and fatal reactions to contrast media: our current understanding	Morcos SK	2005	http://bjr.birjournals.org/cgi/reprint/78/932/686
g)	Prevention of Contrast Media–Induced Nephrotoxicity after Angiographic Procedures	Morcos SK	2005	http://www.jvir.org/aiticle/S1051-0443(07)60593-4/pdf
h)	Prevention of Contrast Media-Induced Nephrotoxicity after Angiographic Procedures	Morcos SK	2005	http://www.jvir.org/aiticle/S1051-0443(07)60593-4/pdf
i)	Contrast-Induced Nephropathy: A Clinical and Evidence-Based Approach	Tepel M *et al.*	2006	http://circ.ahajournals.org/cgi/reprint/113/14/1799
j)	Prophylaxis Strategies for Contrast-Induced Nephropathy	Pannu N *et al.*	2006	http://jama.ama-assn.org/cgi/reprint/295/23/2765
k)	Nephrogenic systemic fibrosis: a serious late adverse reaction to gadodiamide	Thomsen HS	2006	http://www.ismrm.org/special/Editorial%20Eur%20Radiol%20NSF.pdf
1)	Pharmacological prevention of serious anaphylactic reactions due to iodinated contrast media: Systematic review	Tramer MR *et al.*	2006	http://www.bmj.com/cgi/reprint_abr/333/7570/675.pdf
m)	Important Properties of Contrast Media: Focus on Viscosity	Voekz MD *et al.*	2007	http://vasculardiseasemanagement.com/files/docs/GuerbetMarch07JIC.pdf
n)	What nephrologists need to know about gadolinium	Penfield JG Reilly Jr RF	2007	http://www.nature.com/nrneph/journal/v3/nl2/pdf/ncpneph0660.pdf
o)	Contrast-induced nephropathy	Wong GIC, Irwin MG	2007	http://bja.oxfordjournals.Org/cgi/reprint/99/4/474
p)	Contrast media nephropathy—how to diagnose and how to prevent?	Solomon R	2007	http://ndt.oxfordjournals.Org/cgi/reprint/22/7/1812
q)	Gadodiamide-Associated Nephrogenic Systemic Fibrosis: Why Radiologists Should Be Concerned	Broome ER *et al.*	2007	http://www.ajronline.Org/cgi/reprint/188/2/586
r)	lodinated Contrast Media and Their Adverse Reactions	Singh J, Daftrary A	2008	http://tech.snmjournals.org/cgi/reprint/36/2/69
s)	GE Healthcare Position Paper on NSF April 2008	ES Cantor *et al.*	2008	http://images.ctisus.com/GE_whitepaper2.pdf

## End piece

The online database **MR - Technology Information Portal** (MR-TIP.com) was reviewed earlier in the Aug 2007 issue of this journal. Recently, a similar online database covering ultrasonography, titled **UltraSound - Technology Information Portal** is on offer at http://www.us-tip.com/serv1.php?type=ldir.

**US-TIP.com** “is a global information resource aimed at people with professional interests in medical ultrasound technology for imaging and/or therapy.” US-TIP.com, at its home page, links to a US-TIP Resource-Database that contains currently around 1600 entries. Within this are absorbing sections on UltraSound Technology, Knowledge 3D and 4D UltraSound, Contrast Agents, various examinations, History of UltraSound, Image Quality Artifacts, Probes Transducers and UltraSound Physics. Relevant to the topic of this web review, from the above two information portal sites, contrast agents is covered at http://www.mr-tip.com/serv1.php?type=db1anddbs=Contrast%20Agents and http://www.mr-tip.com/serv1.php?type=db1anddbs=Contrast%20Agents and http://www.us-tip.com/serv1.php?type=db1anddbs=Ultrasound%20Contrast%20Agents.

**EduRad** at http://www.edurad.in/index.php is an online initiative aimed at integrating and improving the education of Radiologists by using a professional approach towards the organization of academic programs.Created by Dr. Bharat Parekh, Dr. Deepak Patkar and Dr. Jignesh Thakkar, the portal offers a wide range of web-based education for radiologists. After a mandatory registration, the website offers educative material on Case Reports, Image Gallery, Techno Watch, Classifieds, CME Events, Archives, Teaching Files, Radiology Learning Centre, Online Lectures and Past Conferences (Archives), Live Webcast-Interactive and DMRE (Part-I) lecture series on power point.

A **Wiki** is a special type of web site that enables a group of users to collectively edit, expand, revise, and create content. Wikipedia at http://www.wikipedia.org/ is a free encyclopedia of information created with a notable difference, in that, anyone can edit the information and therefore participate to create an online collaborative reference. What's more, the edited material is assessed for quality and verity by a “behind the scene” moderator group comprising of experts and voluntary professionals.

**Medical Wikis** are the medical equivalent of Wikipedia. They are hosted with an aim to offer comprehensive, veritable and up-to-the-minute information from a single point and that too on a large scale. Medical Wikis are emerging on the net as this List of Medical Wikis at http://davidrothman.net/list-of-medical-wikis/suggests. How far would they evolve and progress, what innovative features would they offer a decade from now, what are the advantageous spinoffs emerging from such websites and what is the endpoint in such websites are some of the hazardous questions that mystifyingly stare at us.

Nevertheless, when it comes to the discipline of radiology, the following Radiology Wikis are available at the moment:

- **RadiologyWiki** at http://www.radiologywiki.org/wiki. This site has a radiology image search functionally similar to ARRS Goldminer, Yottalook and Google image search. Titled Ajax, this filtered radiology image search engine is available at http://www.radiologywiki.org/w/index.php?title=AJAX_Search.
- **RadsWiki** at http://www.radswiki.net/main/index.php?title=Main_Page. This site has many sections, including those on named fractures, radiology in movies, differential diagnosis and a list of signs in radiology.
- **Diagnostic Radiology Wikibooks** is an open-content textbook collection at http://en.wikibooks.org/wiki/Diagnostic_Radiology.
- **wikiRadiography** at http://www.wikiradiography.com/ is an radiography resource on the web with sections on Applied Radiography written by M. J. Fuller, General Radiography, Computed Tomography, U/S, MRI and Mammography Homepages.

